# Self-management program for patients with chronic kidney disease (SMP-CKD) in Southern China: protocol for an ambispective cohort study

**DOI:** 10.1186/s12882-022-02700-2

**Published:** 2022-03-05

**Authors:** Wen-wei Ouyang, Hui-fen Chen, Xue-yi Xu, Xian-long Zhang, Li-zhe Fu, Fang Tang, Ze-huai Wen, Gaetano Marrone, Li-chang Liu, Jing-xia Lin, Xu-sheng Liu, Yi-fan Wu

**Affiliations:** 1 Key Unit of Methodology in Clinical Research, The Second Affiliated Hospital of Guangzhou University of Chinese Medicine (Guangdong Provincial Hospital of Chinese Medicine), Guangzhou, China; 2 Global Health - Health Systems and Policy, Department of Global Public Health, Karolinska Institute, Stockholm, Sweden; 3 The Second Clinical College, Guangzhou University of Chinese Medicine, Guangzhou, Guangdong China; 4 Chronic Disease Management Outpatient, The Second Affiliated Hospital of Guangzhou University of Chinese Medicine (Guangdong Provincial Hospital of Chinese Medicine), Guangzhou, China; 5 Renal Division, Branch court, The Second Affiliated Hospital of Guangzhou University of Chinese Medicine (Guangdong Provincial Hospital of Chinese Medicine), Zhuhai, Guangdong China; 6 Renal Division, Branch court, The Second Affiliated Hospital of Guangzhou University of Chinese Medicine (Guangdong Provincial Hospital of Chinese Medicine), Fangcun District, Guangzhou, Guangdong China; 7 Renal Division, The Second Affiliated Hospital of Guangzhou University of Chinese Medicine (Guangdong Provincial Hospital of Chinese Medicine), Guangzhou, Guangdong China

**Keywords:** Chronic kidney disease, Self-management, Complex intervention, Social cognition theory, Behavior change, Cohort study

## Abstract

**Background:**

Chronic kidney disease (CKD) is a major global health problem. Short-term self-management has been considered to effect some renal and psychological endpoints. However, there are currently very few studies about self-management for CKD that a) have been scientifically designed by a theory-based framework and b) that evaluate the long-term effects and working mechanism. This study presents the rationale and design of a theory-based cohort study to explore how this self-management intervention works and its effectiveness on the Chinese CKD population.

**Methods:**

In this ambispective intervention cohort study,1,200 patients with CKD stages 1–5 will be recruited from July 2015 to July 2024 in 3 branches of Guangdong Provincial Hospital of Chinese Medicine (GPHCM) in Guangdong province, China. The patients in the self-management cohort will choose to receive an intervention that consists of education, nutrition/diet modification, lifestyle change recommendation, medication review, and psychology support based on Social Cognition Theory (SCT). The patients in the control cohort will do regular follow-ups based on the clinic rules. All the patients will be followed up for 5 years, or until the occurrence of a primary outcome. Detailed clinical, laboratory markers, nutritional status, psychological exposures and outcome questionaries will be collected semiannually in CKD stage 1–2 and trimonthly in stage 3–5 patients. The primary outcome is the occurrence of composite clinical endpoints (doubling of serum creatinine level, ESKD, loss of renal function (≥ 40% decline in GFR from baseline), death, major cardiovascular or cerebrovascular events). The main secondary outcomes include the absolute change and slope of eGFR, absolute changes of urinary protein creatinine ratio, 24-h urine proteinuria, intact parathyroid hormone level, and self-management adherence rate and quality of life from baseline to end of the study. The effectiveness of self-management will be analyzed and the association between longitudinal trajectories of self-management and renal outcomes will be evaluated.

**Discussion:**

This study aims to provide further evidence for the effectiveness of theory-based self-management in CKD patients and to improve the lives of patients with CKD by slowing progression, improving psychological well-being and overall quality of life.

**Trial registration:**

Chinese Clinical Trial Register (ChiCTR1900024633). 19 July, 2019. http://www.chictr.org.cn/showproj.aspx?proj=38378

## Background

Chronic kidney disease (CKD) is a serious global public health problem. It is reported a prevalence rate of 9.1% in global and 10.8% in Mainland China adult population [1, 2]. CKD can cause numerous complications, high morbidity, increased mortality and heavy economic burden especially progress to the end-stage kidney disease (ESKD) [3, 4]. Blood pressure control with angiotensin-converting enzyme inhibitors (ACEi) or angiotensin receptor blockers (ARB) is considered the main pharmic way to prevent CKD progression [5]. However, the traditional treatment method involves many challenges, such as insufficient knowledge acquired by patients, low participation in disease treatment decision-making, and low compliance in the treatment [6]. Hence, finding a new strategy for slow CKD progression is urgent.

Self-management, defined as the active management by individuals of their treatment, symptoms, lifestyle and the physical and psychological consequences inherent in living with a chronic condition, is an established treatment for managing CKD in recent years [7–12]. It has contributed to a paradigm shift from a paternalistic model to a more equitable and collaborative model between nephrologists and patients [13, 14]. Dietary adjustment is an important component of the self-management intervention due to disordered nutritional status and commonly appeared protein-energy wasting condition in the CKD population [15]. A scoping review revealed that diet/nutrition intervention accounts for over 60% of all list self-management topics [16]. A nutrition intervention, together with patient’s education, lifestyle change, risk factors control, psychological support, pharmacist medication review, etc. is recommended as preferred non-pharmacological alternative strategies for managing CKD patients [5].

Studies showed this emerging strategy with the function to relieve renal-related symptoms, enhance patient-centeredness and decrease patients’ concerns with depression, anxiety, self-perceived burdens [5, 15, 17–20]. However, the evidence of self-management for renal outcomes is contradictory. A randomized controlled trial (RCT) in Taiwan showed self-management may slow the reduction of estimated glomerular filtration rate (eGFR) in late-stage CKD patients within 12 months follow up, it suggested that self-management may become an effective strategy for managing CKD patients, but systematic review and meta-analysis in RCT have shown that although self-management interventions can lower 24-h urinary protein excretion, improve self-care activities and systolic blood pressure than usual care, it did not provide additional benefits for eGFR and other renal outcomes in non-dialysis patients [17–20]. While considering the median follow-up time of these studies is 13.44 months, in such a short follow-up period, it may be difficult to distinguish the real effects of self-management [20]. Furthermore, self-management is not easy to evaluate, especially in the long-term study. Adherence to the intervention plays a crucial role in the effectiveness of nonpharmacologic treatment strategies [21]. But self-management has low adherence rates, and long-term persistence is difficult [22]. During the study period, most patients’ clinical status and their participation in self-management changed dynamically, which can lead to inaccurate measures of interventions and get biased research conclusions. Therefore, its effect on thwarting the progression of CKD and other outcomes still needs further research.

What’s more, self-management interventions can be seen as complex interventions, it means not only number of and interactions between components within the interventions, but also the complexity of how the intervention works [23]. However, many studies give only a conceptual or general definition of self-management interventions, or no definition at all [18–20, 24]. When the variety among self-management interventions is not taken into account and no clear operational definition is posited, this might lead to incorrect conclusions [25, 26]. Hence, a theory-based model is preferred, it can causally link behavioral determinants, through behavior, to physiological and biochemical variables, and health outcomes [27]. This theoretical basis approach ensures interventions have a greater chance of being effective, and the reasons for these effects can be deduced [28]. However, existing research showed only 20% of studies were designed based on a theoretical framework. More knowledge about how self-management will work for CKD is still need to be studied [16].

Considering the problems and gaps above, Self-Management Program for Patients with Chronic Kidney Disease Cohort (SMP-CKD cohort), a theory-based intervention cohort study alongside a process evaluation aiming to improve the lives of patients with CKD by slowing progression and improving overall Quality of Life (QoL) will be performed. In this protocol, we present the rationale and design of this ongoing hospital-based cohort study. The objective of this cohort include i) to evaluate the long-term effectiveness of the theory-based self-management program in the Chinese CKD population; ii) to assess its longitudinal relationship to surrogate markers and renal outcomes, and; iii) to explore the working mechanism of this complex intervention.

## Methods

### Study design and study population

An ambispective cohort study will be conducted in the department of nephrology at the three branches of Guangdong Provincial Hospital of Chinese Medicine (GPHCM) in Guangdong province, China. Two branches are located in different Districts in Guangzhou city (Yuexiu and Fangcun District) and one in Zhuhai city. The study population includes all CKD 1–5 patients (speak Cantonese or Mandarin) attending the CKD consultation outpatient clinic in GPHCM from July 2015 to July 2024. Of 200 CKD patients were already registered in the retrospective cohort from July 2015 to July 2019 and they will keep following in the prospective cohort. Beginning in August 2019, a prospective cohort will be built on. All the patients will be followed for at least 5 years, or until the occurrence of a primary outcome.

CKD is defined by a glomerular filtration rate (GFR) < 60 mL/min/1.73 m2 or markers of kidney damage, or both, of at least 3 months duration [5]. GFR is calculated by CKD-EPI equation, and CKD staging will use the Kidney Disease Outcomes Quality Initiative (KDOQI) definition [29]. Patients are eligible for inclusion if they are Chinese, aged 18 to 80 years, have CKD (Stages 1–5), and give a written signed informed consent. Exclusion criteria for the study are psychosis or unable to cooperate with clinical staff for other reasons and have a history of dialysis or renal transplant.

### Screening and Enrollment

**Fig. 1:**
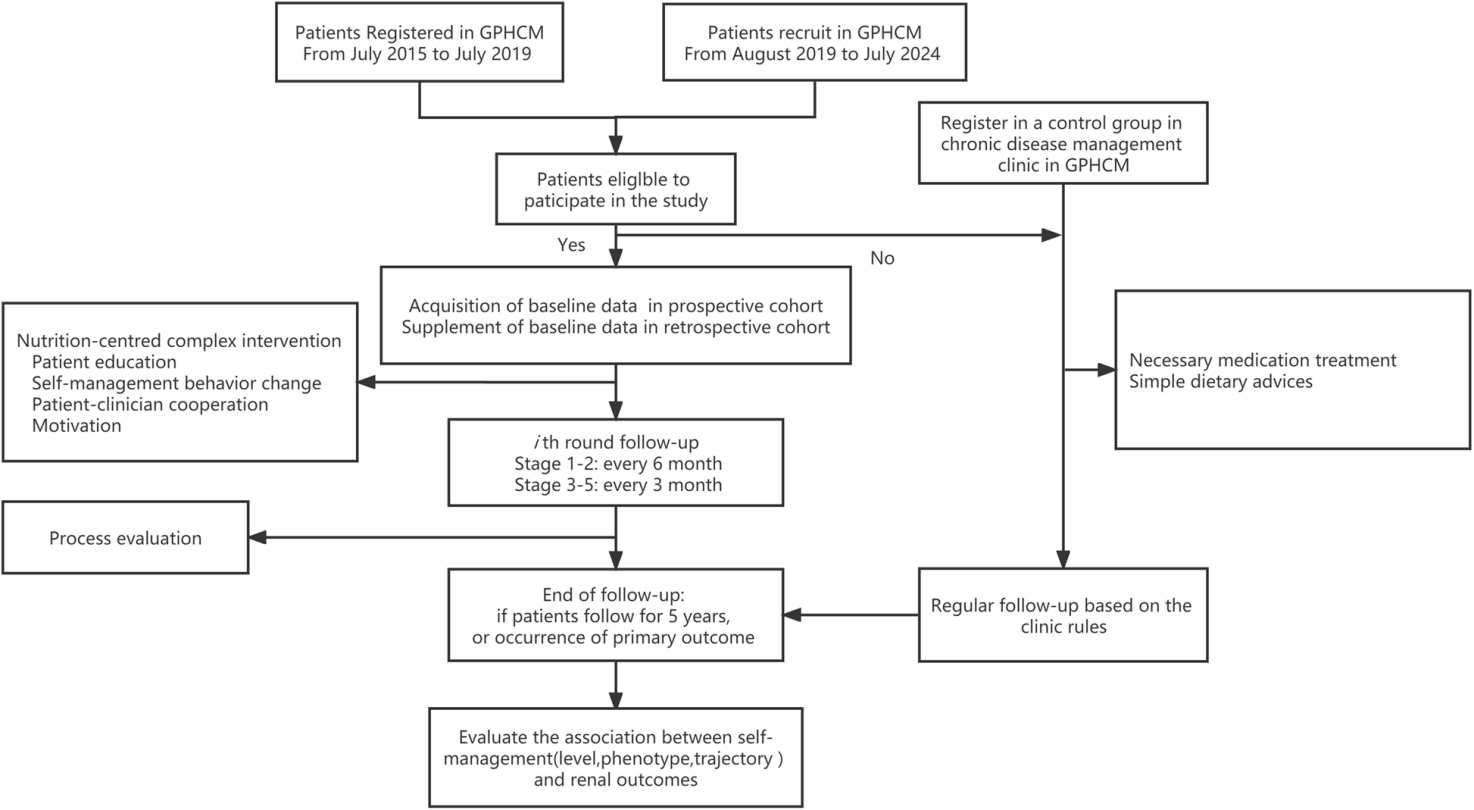
Details of study process

Research assistants in our team are responsible for identifying patients from retrospective cohort and consultation outpatient in the Department of Chronic Disease Management at GPHCM to ensure they meet inclusion criteria. Considering the retrospective cohort is an observational cohort, this study will use the date when the patients are enrolled in the new cohort as their baseline time. After pre-evaluation, nurses will first make an appointment and then explain the study to candidates through a face-to-face interview and answer any questions they have. If the patients do not agree to enroll in SMP-CKD cohort, they will be registered in the chronic disease management clinic in GPHCM and do regular follow-up based on the clinic rules (as the control cohort). After confirming eligibility, all participants will sign an informed consent. (Study process can be found in Fig. 1).

### Follow-up and Retention

After the pre-evaluation of the baseline variables, a corresponding individualized follow-up calendar will be formulated. If there are no special circumstances, visits will be conducted semiannually in stage 1–2 patients and trimonthly in stage 3–5 CKD patients. Flexibility of 1 week before or after the next visit is allowed. The timeline can be found in Table 1.

**Table 1:**
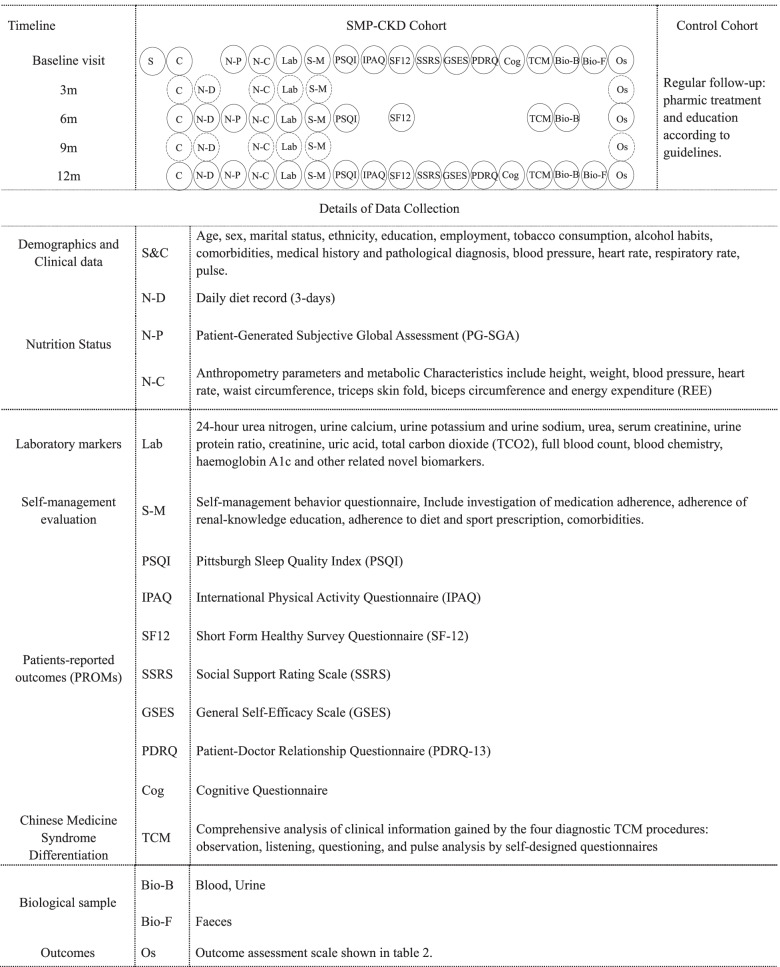
Details about data collection

Items that all the patients should finish; Extra items that patients with CKD stage 3–5 should finish

In view of reducing the systematic attrition in this long-term cohort, some retention strategies will be considered: 1) During each visit, patients will receive a paper-version calendar for reminding their next clinic visit. 2) A week before a visit, nurses in the research team will send text messages to remind them the date for their appointment. Then, those who do not respond to the text messages will be contacted again within 3 days. 3) A final notice will be sent to patients via social media apps-WeChat, a very popular communication tool in China to keep them informed of the appointment. 4) Gift/ freebies incentives for finishing a one-year follow-up circle.

Self-management intervention design and process evaluation.

### Intervention design

Social Cognition Theory (SCT) will be applied to design a theory-based complex intervention. According to SCT, motivational enhancements can ultimately lead to behavior modification. It can help the individual modify their behaviors, and then elevate the individual’s perceived self-efficacy and outcome expectations [30]. The Behavior Change Technique Taxonomy v1 will be used to specify the self-management intervention functions and behavior change techniques [31].

Self-management interventions components will be selected by reviewing existing epidemiological evidence, consulting clinical psychologists, sociologist, epidemiologists, and holding group meetings. The components of this complex intervention include education, nutrition/diet modification, lifestyle change recommendation, medication review and psychology support. A multidisciplinary team consisting of nephrologists, nurses, dietitians, and postgraduate students will be in charge of the delivery of all the interventions. All the team members will participate in a 1-day workshop to standardize their delivery.

The education will entail a 1.5-h integrated slide-lecture, delivered by nephrologists and dietitians trimonthly involving general CKD knowledge, dietary management, healthy lifestyle, pharmacological regimens and Chinese herbal medicine instructions. Cooking courses will be offered semiannually for 2 h a session with different topics (macro-and micro-nutrients intake and restriction, label reading, eating out, et al.), provided by dietitians and nurses. All the courses will be taught in Cantonese or Mandarin to accommodate the main languages spoken in the Guangdong Province.

After assessing baseline anthropometric parameters and biochemical findings, patients will receive face-to-face dietary counseling from a dietitian includes the protein, caloric, potassium, phosphorus, sodium intake recommendations. Before each follow-up/clinic visit, they will be asked to fill a diet diary to record all foods and beverages consumed for 72 h, in succession two weekdays and one weekend day, then dietitian will assess their compliance by analyzing the diary data and offer new individualized dietary prescriptions. All these recommendations and prescriptions are based on the Chinese guideline [32].

During each visit, assessments include questionaries review of CKD knowledge, symptoms, comorbidities, lifestyle change (physical activity attendance, smoking status, alcohol intake), psychological and self-efficacy status by postgraduate students and nurses will be carried out. Then nephrologist will hold a 30-min one-on-one, face-to-face individualized interview based on current self-management practices and biochemical markers. The content of the interview will cover medication review, knowledge gain, thoughts and feelings, using an interactive, psychosocial approach underpinned by the SCT.

Some behavior change techniques will be used in this study, they can be grouped into 5 domains: goals and planning, feedback and monitoring, social support, self-belief and shaping knowledge. On the goals and planning domain, the research team set some individualized goals in terms of the behavior to be achieved (e.g., daily walking at least 60 min), or a goal for a positive outcome at the beginning of the study. In the next follow-up, we will review behavior goals jointly with patients and consider modifying goals in light of their achievement. In the feedback and monitoring domain, self-monitoring of behavior will be adopted (e.g., 3-day food diary), then the research team monitor and provide evaluative feedback on the performance of patients’ behavior (e.g., check how many nutrients they intake each day by analyzing their food diary) or biofeedback (e.g., inform their blood pressure or eGFR slope curve). The techniques of instruction on how to perform a behavior and information about antecedents will be applied to the shaping knowledge domain. For example, when conducting nutrition-related lectures, food models will be displayed. Both practical and emotional social support will be used in this study, verbal persuasion about capability and mental rehearsal of successful performance techniques will be used to boost self-efficacy.

Medical staff, patients and their caregivers are included in the intervention to facilitate their behavior change. To encourage a collaborative patient role, the research team will encourage patients to report problems directly to team members whenever they raise questions, through face-to-face, telephone and WeChat apps. Details are shown in Table 2 logic model for self-management in CKD.

**Table 2:**
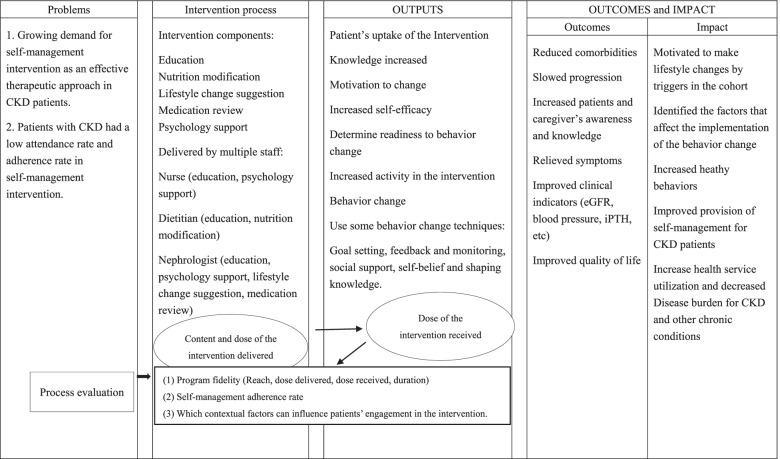
Logic Model for Self-management in CKD

### Process evaluation

There are 3 aims of this process evaluation (1) program fidelity between the planned and actual implementation of the intervention (2) self-management adherence rate (3) contextual factors influencing and maintaining user engagement with the intervention. The details are shown in Table 3.

**Table 3:** Process Evaluation components and Definition

Components	Definition	Data source
Program fidelity		
Reach	Proportion of intended target patients participate in the intervention	Delivery records
Dose delivered	The amount of the intervention components provided to patients	Delivery records, Audio records
Dose received	What extent participants actively engaged with and/or used the materials provided to them	Delivery records, Cognitive questionnaire
Duration	How long the patients participate in the intervention	Delivery records
Contextual factors	Explore contextual factors affecting patients’ engagement in the intervention	Patient survey Patient characteristics SSRS, GSES, PDRQ scale
Adherence	Self-management intervention adherence rate of the patients	Delivery records Self-management behavior questionnaire Diet diary review

An independent nurse team will be responsible for process evaluation, including data collection and management. This process evaluation team will design the process evaluation program, conduct the evaluation, and monitor its implementation. The process evaluation data will be analyzed before analyzing the cohort data.

Note: SSRS, Social Support Rating Scale; GSES, General Self-Efficacy Scale; DPRQ, Patient-Doctor Relationship Questionnaire.

## Measures

### Exposure measures

The details of the data collection are shown in Table 1. Social-demographic and clinical data will be collected only in prospective cohort by a predetermined questionnaire during the initial visit. Physical examination and laboratory markers will be collected trimonthly in patients with CKD 3–5 and semiannually in patients with CKD 1–2.

The nutritional parameters will be evaluated by 1) a validated daily diet record form recording all foods and beverages consumed for 72 h, in succession two weekdays and one weekend day before the 24-h urine sample is collected. Trained staff will teach all patients how to keep proper records in their diet logs. These dietary data will be converted into daily nutrient intakes by using the “Chinese food composition list, 2nd edition” [33]. 2) Inbody-770 and tool measuring for anthropometric parameters and Medgraphics Ultima CCM for metabolic characteristics [34, 35]. 3) Patient-Generated Subjective Global Assessment (PG-SGA) for symptoms, metabolic stress and nutritional status [36].

A 11-item self-management behavior questionnaire developed by our team will be used to measure the self-management compliance. Patients-reported outcomes (PROMs) will be used to identify lifestyle factors, self-efficacy, psychological status and quality of life. A cognitive questionnaire will be given to determine patients’ cognitive levels so that the nursing group can schedule receptive and individualized courses [37]. Social support score will be collected by the Social Support Rating Scale (SSRS), self-efficacy score will be assessed by General Self-Efficacy Scale (GSES), and patient and doctor relationship will be measured by a developed Patient-Doctor Relationship Questionnaire (PDRQ-13) [38–40]. Patients’ other lifestyle factors (e.g., sleep and physical activity) will be evaluated by the Pittsburgh Sleep Quality Index (PSQI), and the International Physical Activity Questionnaire (IPAQ) [41, 42]. The Short Form Healthy Survey Questionnaire (SF-12) will be used to measure CKD patients’ quality of life [43]. All above self-reported questionnaires are validated in Chinese population.

### Outcome measures

The primary outcome is the occurrence of composite clinical endpoints (doubling of serum creatinine level, ESKD(eGFR < 15 ml/min for CKD 1–4 patients; or dialysis for at least 30 days or kidney transplant for patients with CKD 1–5), loss of renal function(≥ 40% decline in GFR from baseline), death(from any cause), major cardiovascular or cerebrovascular events) during the 5-year follow up (shown in Table 4) [44, 45]. Those outcome data will be collected through the outcome assessment scale during each visit by reviewing the laboratory test results and the medical record in Hospital Information System (HIS).

**Table 4:** Definition of composite clinical endpoints

Clinical events	Definition
1.A doubling of serum creatinine levels	Doubling of the serum creatinine level from baseline sustained for at least 30 days according to central laboratory assessment
2.End-stage kidney disease (ESKD)	eGFR < 15 ml/min for CKD 1–4 patients; or dialysis or kidney transplant for CKD 1–5 patients
3.Loss of renal function	≥ 40% decline in GFR from baseline
4.Death	Death from any cause
5.Major cardiovascular or cerebrovascular events	Acute heart failure, myocardial infarction, ischemic cerebral infarction or intracranial hemorrhage

The main secondary outcomes include the absolute change and slope of eGFR, absolute changes of urinary protein creatinine ratio, 24-h urine proteinuria, intact parathyroid hormone level (iPTH), and self-management adherence rates (evaluated by self-management behavior questionnaire) and quality of life. Other outcomes include serum creatinine level (SCr), blood urea nitrogen level (BUN), plasma albumin, triglyceride, cholesterol, calcium, phosphate and other Laboratory markers and cardiovascular function.

## Sample size calculation

We selected the occurrence of composite endpoints as the primary study outcomes to calculate the sample size. Previous studies have reported that the composite endpoints rate range from 18.1–126/1,000 person-years [46, 47]. Based on these studies and our previous data, we estimated that the incidence rate of a composite endpoint in our cohort would be 60/1,000 person-years. There are about 30 variables that may affect prognosis, and there should be at least 10 endpoints per variable. Thus, we anticipate that recruiting 1,200 participants with 300 events over a 5-year follow-up will ensure adequate statistical power.

## Statistical analysis plan

Categorical demographic and other baseline characteristics variables will be summarized by frequencies and percentages. Continuous variables will be summarized by both mean and standard deviation for data with normal distributions or median and inter-quartile range for non-normally distributed data. Either a Chi-square or Fisher’s exact test will be used to compare categorical variables between groups, while Mann–Whitney U-test or Student’s t-test will be used for continuous variables, if necessary.

The primary objective of this cohort is to evaluate the effectiveness of the self-management program, we plan to perform three separate analyses for the primary outcome. First, the incidence rate of composite endpoint events will be analyzed by the Chi-squared test. Second, time to occurrence of composite clinical endpoints will be analyzed using log rank test. The hazard ratio (HR) with 95% CI will be estimated by using the Cox proportional hazards model or competitive risk model. Third, growth mixture models and group-based trajectory models (GBTM) will be used to analyze the trajectories of exposure and outcome variables [48]. Covariates for the models will be selected based on prior knowledge and published papers. Logistic regression or Poisson regression will be used to analyze categorical outcomes. Liner mixed-effect model will be applied to the longitudinal data, such as the absolute change, and slope of eGFR and other secondary outcomes.

Even with some retention strategies, considerable missing data is expected over 5-years follow-up. The proportion of missing data will be summarized in each group and at each visit point. If there is less than 20% of data missing for the covariate data, we will perform a complete case analysis. If there is more than 20% of missing data, we will perform Little’s test and use multiple imputations under the assumption of missing is at random. Subgroup analyses will be conducted across baseline eGFR levels, demographic status and other factors, if possible. To test the credibility and robustness of the findings, several sensitivity analyses will be performed, including E-value and other advanced models [49]. Effect modification will be investigated using stratified analyses and formal tests of interaction. Mediation will be analyzed by adding the hypothesized mediators, one by one, to the confounder-adjusted models to study the extent to which they explain any association found.

All analysis will be performed in PASW 18.0 (SPSS Inc. IBM Corporation, Armonk, New York, USA) and STATA SE/15.0 (Stata Corp. College Station, Texas, USA).

## Discussion

To the best of our knowledge, this is the first theory-based self-management cohort conducted in Mainland China for CKD patients. This paper presents the protocol for the design and process evaluation of this self-management cohort. We believe there are several elements in the design and implementation of this study that allow advantages for quality data to inform the management of CKD in China.

Previous studies have regarded self-management as a plausible way to slow CKD progression [20]. However, due to the short follow-up time, their results have certain limitations. This is because self-management adherence is high in these studies, but patients experience ‘active’ self-management initially whereas self-management behavior is neglected as time progresses [50]. Meanwhile, applying self-management to CKD management needs flexible appointments and re-planning options between nephrologists and patients which is more suitable to achieve by cohort research. Therefore, it is worthwhile to study the effect of self-management through a long-term follow-up in a real-world medical environment.

However, it’s not easy to maintain patients’ compliance in the long-term follow-up study. Results from other studies showed theory-based intervention can improve medication and self-management adherence in other chronic conditions [51, 52]. Guidelines also recommend the explicit use of behavior change for addressing lifestyle risk factors when designing and reporting interventions for patients with CKD [53, 54]. In this cohort, behavior change is based on the SCT framework [55]. The application of this theory has shown positive effects on health behaviors in many chronic disease settings such as diabetes, heart disease and neurological disorders [56, 57]. According to this theory, this cohort study will determine the specific items to personalize educational content and behavior change strategies based on psychosocial determinants of self-efficacy, self-regulation, skill mastery, and outcome expectations. Health education and self-management programs focus not only on changing the patient’s awareness of the disease, but also on improving their personalized self-efficacy attainment strategies addressing motivation to change motivational enhancement strategies, behavior modification, and emotion management and stress management issues [58, 59].

Since self-management is a complex intervention, how to deliver a reasonable self-management intervention is still being explored [60]. We use process evaluation to provide novel information and understanding about how self-management works in clinical practice. Process evaluation will evaluate the components of execution, the influence mechanism and context to identify and understand the core components of self-management [61]. This not only can increase understanding of how the process is delivered, but also help us derive more reliable conclusions about the effectiveness of interventions in terms of whether they can be accepted by patients and healthcare professionals [62].

However, this study has some limitations. Firstly, potential selection bias or confounder effect may exist because it is a hospital-based design study, the results may not sufficiently generalize to other populations. But we try to select 3 branches hospitals, while the source populations of these three hospitals have different demographic and socioeconomic characteristics, it can increase the confidence in the generalizability of the study. And more so than other cohorts, its hospital-based design can ascertain that CKD patients’ diagnoses and the interpretations of the renal outcomes are accurate which means the risk of misclassification bias is low [63]. Secondly, the long study period is a double-edged sword. Although a considerable amount of follow-up data can be obtained during the study which allows us to have the possibilities to dynamically evaluate patients’ condition and study the relationship among longitudinal trajectories of exposure, surrogate markers, and renal outcomes [48]. High rates of patient's lost-to-follow-up are inevitable, how to minimize the drop-outs and deal with the missing data is a big challenge. One solution is the patients enrolled in this cohort are registered in the chronic disease management clinic in GPHCM, they will return to the outpatient clinic for regular checkups because we are the designated hospital for patients' medical insurance. The other solution is some retention strategies have been adopted, such as reducing barriers to participation, setting reminder strategies and gift/freebies incentives.

In a word, although self-management has been recommended in CKD guidelines, it is still in its infancy in China. Better knowledge about the topic may highlight the need for CKD patients and healthcare professionals to facilitate lifestyle change, manage symptoms, and offer rational support. The results of this project are expected to provide evidence of high methodological quality on the effectiveness of self-management intervention for CKD patients in order to improve the lives of patients with CKD by slowing progression, improving psychological well-being and overall quality of life.

## Data Availability

This study was conducted in the nephrology department at Guangdong Provincial Hospital of Chinese Medicine (GPHCM). All of the data are available in GPHCM’s outpatient system. The datasets used and analysed during the current study available from the corresponding author on reasonable request.

## Abbreviations

CKD: Chronic kidney disease
ESRD: End-stage kidney disease
ACEi: Angiotensin-converting enzyme inhibitors
ARB: Angiotensin receptor blockers
RCT: Randomized controlled trial
eGFR: Estimated glomerular filtration rate
SMP-CKD cohort: Self-Management Program for Patients with Chronic Kidney Disease Cohort
QoL: Quality of Life
GPHCM: Guangdong Provincial Hospital of Chinese Medicine
KDOQI: Kidney Disease Outcomes Quality Initiative
SCT: Social Cognition Theory
PG-SGA: Patient-Generated Subjective Global Assessment
PROMs: Patients-reported outcomes
SSRS: Social Support Rating Scale
GSES: General Self-Efficacy Scale
PDRQ-13: Patient-Doctor Relationship Questionnaire
PSQI: Pittsburgh Sleep Quality Index
IPAQ: International Physical Activity Questionnaire
SF-12: Short Form Healthy Survey Questionnaire
HIS: Hospital Information System
iPTH: Intact parathyroid hormone level
SCr: Serum creatinine level
BUN: Blood urea nitrogen level
HR: Hazard ratio
GBTM: Group-based trajectory models

## References

[1] Global, regional, and national burden of chronic kidney disease (2020). 1990–2017: a systematic analysis for the Global Burden of Disease Study 2017. Lancet.

[2] Zhang L, Wang F, Wang L, Wang W, Liu B, Liu J (2012). Prevalence of chronic kidney disease in China a cross-sectional survey. Lancet.

[3] Dong Y, Qu X, Wu G, Luo X, Tang B, Wu F (2019). Advances in the Detection, Mechanism and Therapy of Chronic Kidney Disease. Curr Pharm Des.

[4] Webster AC, Nagler EV, Morton RL, Masson P (2017). Chronic Kidney Disease. Lancet.

[5] Inker LA, Astor BC, Fox CH, Isakova T, Lash JP, Peralta CA (2014). KDOQI US commentary on the 2012 KDIGO clinical practice guideline for the evaluation and management of CKD. Am J Kidney Dis.

[6] Keith DS, Nichols GA, Gullion CM, Brown JB, Smith DH (2004). Longitudinal follow-up and outcomes among a population with chronic kidney disease in a large managed care organization. Arch Intern Med.

[7] Oh TR, Choi HS, Kim CS, Bae EH, Oh YK, Kim YS (2019). Association between health related quality of life and progression of chronic kidney disease. Sci Rep.

[8] McCusker J, Lambert SD, Haggerty J, Yaffe MJ, Belzile E, Ciampi A (2019). Self-management support in primary care is associated with improvement in patient activation. Patient Educ Couns.

[9] van Eikenhorst L, Taxis K, van Dijk L, de Gier H (2017). Pharmacist-Led Self-management Interventions to Improve Diabetes Outcomes A Systematic Literature Review and Meta-Analysis. Front Pharmacol.

[10] Welch JL, Johnson M, Zimmerman L, Russell CL, Perkins SM, Decker BS (2015). Self-management interventions in stages 1 to 4 chronic kidney disease: an integrative review. West J Nurs Res.

[11] Devins GM, Mendelssohn DC, Barre PE, Binik YM (2003). Predialysis psychoeducational intervention and coping styles influence time to dialysis in chronic kidney disease. Am J Kidney Dis.

[12] Lorig KR, Holman H (2003). Self-management education: history, definition, outcomes, and mechanisms. Ann Behav Med.

[13] Aujoulat I, Marcolongo R, Bonadiman L, Deccache A (2008). Reconsidering patient empowerment in chronic illness: a critique of models of self-efficacy and bodily control. Soc Sci Med.

[14] Kawamoto K, Houlihan CA, Balas EA, Lobach DF (2005). Improving clinical practice using clinical decision support systems: a systematic review of trials to identify features critical to success. BMJ.

[15] Kalantar-Zadeh K, Fouque D (2017). Nutritional Management of Chronic Kidney Disease. N Engl J Med.

[16] Donald M, Kahlon BK, Beanlands H, Straus S, Ronksley P, Herrington G (2018). Self-management interventions for adults with chronic kidney disease: a scoping review. BMJ OPEN.

[17] Chen SH, Tsai YF, Sun CY, Wu IW, Lee CC, Wu MS (2011). The impact of self-management support on the progression of chronic kidney disease–a prospective randomized controlled trial. Nephrol Dial Transplant.

[18] Lee MC, Wu SV, Hsieh NC, Tsai JM (2016). Self-Management Programs on eGFR, Depression, and Quality of Life among Patients with Chronic Kidney Disease: A Meta-Analysis. Asian Nurs Res (Korean Soc Nurs Sci).

[19] Zimbudzi E, Lo C, Misso ML, Ranasinha S, Kerr PG, Teede HJ (2018). Effectiveness of self-management support interventions for people with comorbid diabetes and chronic kidney disease: a systematic review and meta-analysis. Syst Rev.

[20] Peng S, He J, Huang J, Lun L, Zeng J, Zeng S (2019). Self-management interventions for chronic kidney disease: a systematic review and meta-analysis. BMC Nephrol.

[21] Inouye SK, Bogardus SJ, Williams CS, Leo-Summers L, Agostini JV (2003). The role of adherence on the effectiveness of nonpharmacologic interventions: evidence from the delirium prevention trial. Arch Intern Med.

[22] Mathes T, Jaschinski T, Pieper D (2014). Adherence influencing factors - a systematic review of systematic reviews. Arch Public Health.

[23] Craig P, Dieppe P, Macintyre S, Michie S, Nazareth I, Petticrew M (2008). Developing and evaluating complex interventions: the new Medical Research Council guidance. BMJ.

[24] Lin MY, Liu MF, Hsu LF, Tsai PS (2017). Effects of self-management on chronic kidney disease: A meta-analysis. Int J Nurs Stud.

[25] Jonkman NH, Schuurmans MJ, Jaarsma T, Shortridge-Baggett LM, Hoes AW, Trappenburg JCA (2016). Self-management interventions: Proposal and validation of a new operational definition. J Clin Epidemiol.

[26] Bourbeau J, van der Palen J (2009). Promoting effective self-management programmes to improve COPD. Eur Respir J.

[27] Hardeman W, Sutton S, Griffin S, Johnston M, White A, Wareham NJ (2005). A causal modelling approach to the development of theory-based behaviour change programmes for trial evaluation. Health Educ Res.

[28] Heath G, Cooke R, Cameron E (2015). A Theory-Based Approach for Developing Interventions to Change Patient Behaviours: A Medication Adherence Example from Paediatric Secondary Care. Healthcare (Basel).

[29] Inker LA, Shaffi K, Levey AS (2012). Estimating glomerular filtration rate using the chronic kidney disease-epidemiology collaboration creatinine equation: better risk predictions. Circ Heart Fail.

[30] Bandura A (2004). Health promotion by social cognitive means. Health Educ Behav.

[31] Michie S, Richardson M, Johnston M, Abraham C, Francis J, Hardeman W (2013). The behavior change technique taxonomy (v1) of 93 hierarchically clustered techniques: building an international consensus for the reporting of behavior change interventions. Ann Behav Med.

[32] National Health and Family Planning Commission of China. Dietary guide for chronic kidney disease patients. 2017.WS/T557—2017.

[33] Yue-xin Yang, Guang-ya Wang, Xing-chang Pan. China Food Composition [Book 1.2nd Edition], Beijing Medical University Press. 2009.

[34] InBody Europe BV. www.inbody.de. Accessed 29 Oct 2021.

[35] MGC Diagnostics. http://www.mgc-china.com/maijiafei/products/10448997.html. Accessed 29 Oct 2021.

[36] Bauer J, Capra S, Ferguson M (2002). Use of the scored Patient-Generated Subjective Global Assessment (PG-SGA) as a nutrition assessment tool in patients with cancer. Eur J Clin Nutr.

[37] Peng S, He J, Huang J, Tan J, Liu M, Liu X (2019). A chronic kidney disease patient awareness questionnaire: Development and validation. Plos One.

[38] Xiao SY. Social support rating scale. Chinese Mental Health Journal. 1993 1993–01–01:42–6.

[39] Cheung SK, Sun SY (1999). Assessment of optimistic self-beliefs: further validation of the Chinese version of the General Self-Efficacy Scale. Psychol Rep.

[40] Hui Yang. Developing and Evaluating PDRQ and DDPRQ in Chinese Version—Quantitative Research on Physician-Patient Relationship. Shanxi Medical University. 2011. https://kns.cnki.net/KCMS/detail/detail.aspx?dbname=CMFD2011&filename=1011092368.nh

[41] Buysse DJ, Reynolds CR, Monk TH, Berman SR, Kupfer DJ (1989). The Pittsburgh Sleep Quality Index: a new instrument for psychiatric practice and research. Psychiatry Res.

[42] Craig CL, Marshall AL, Sjostrom M, Bauman AE, Booth ML, Ainsworth BE (2003). International physical activity questionnaire: 12-country reliability and validity. Med Sci Sports Exerc.

[43] Lam CL, Tse EY, Gandek B (2005). Is the standard SF-12 health survey valid and equivalent for a Chinese population?. QUAL LIFE RES.

[44] Levey AS, Inker LA, Matsushita K, Greene T, Willis K, Lewis E (2014). GFR decline as an end point for clinical trials in CKD: a scientific workshop sponsored by the National Kidney Foundation and the US Food and Drug Administration. Am J Kidney Dis.

[45] Perkovic V, Jardine MJ, Neal B, Bompoint S, Heerspink H, Charytan DM (2019). Canagliflozin and Renal Outcomes in Type 2 Diabetes and Nephropathy. N Engl J Med.

[46] Ricardo AC, Anderson CA, Yang W, Zhang X, Fischer MJ, Dember LM (2015). Healthy lifestyle and risk of kidney disease progression, atherosclerotic events, and death in CKD: findings from the Chronic Renal Insufficiency Cohort (CRIC) Study. Am J Kidney Dis.

[47] Inaguma D, Imai E, Takeuchi A, Ohashi Y, Watanabe T, Nitta K (2017). Risk factors for CKD progression in Japanese patients: findings from the Chronic Kidney Disease Japan Cohort (CKD-JAC) study. Clin Exp Nephrol.

[48] Herle M, Micali N, Abdulkadir M, Loos R, Bryant-Waugh R, Hubel C (2020). Identifying typical trajectories in longitudinal data: modelling strategies and interpretations. Eur J Epidemiol.

[49] VanderWeele TJ, Ding P (2017). Sensitivity Analysis in Observational Research: Introducing the E-Value. Ann Intern Med.

[50] van Smoorenburg AN, Hertroijs D, Dekkers T, Elissen A, Melles M (2019). Patients' perspective on self-management: type 2 diabetes in daily life. Bmc Health Serv Res.

[51] Patton DE, Hughes CM, Cadogan CA, Ryan CA (2017). Theory-Based Interventions to Improve Medication Adherence in Older Adults Prescribed Polypharmacy: A Systematic Review. Drugs Aging.

[52] Menti D, Limbert C, Lyrakos G. Investigating the effectiveness of theory-based interventions for improving treatment adherence of patients with type 2 Diabetes Mellitus: A systematic review of Randomised Controlled Clinical Trials. 2019 2019–01–01.

[53] National IFHA. Behaviour change: individual approaches.: National Institute for Health and Care Excellence (NICE); 2014.

[54] Royal ACOG. Guidelines for preventive activities in general practice.: Royal Australian College of General Practitioners; 2016.

[55] Bandura A (2004). Health promotion by social cognitive means. Health Educ Behav.

[56] Aljasem LI, Peyrot M, Wissow L, Rubin RR (2001). The impact of barriers and self-efficacy on self-care behaviors in type 2 diabetes. Diabetes Educ.

[57] Sajatovic M, Colon-Zimmermann K, Kahriman M, Fuentes-Casiano E, Liu H, Tatsuoka C (2018). A 6-month prospective randomized controlled trial of remotely delivered group format epilepsy self-management versus waitlist control for high-risk people with epilepsy. Epilepsia.

[58] Norris SL, Lau J, Smith SJ, Schmid CH, Engelgau MM (2002). Self-management education for adults with type 2 diabetes: a meta-analysis of the effect on glycemic control. Diabetes Care.

[59] Funnell MM, Anderson RM (2004). Empowerment and Self-Management of Diabetes. Clinical Diabetes.

[60] Schrauben SJ, Hsu JY, Rosas SE, Jaar BG, Zhang X, Deo R (2018). CKD Self-management: Phenotypes and Associations With Clinical Outcomes. Am J Kidney Dis.

[61] Richards, D.A., Rahm Hallberg, I. (Eds.). Complex Interventions in Health: An overview of research methods [Book 1st Edition], Routledge. 2015. 10.4324/9780203794982

[62] Nyberg A, Wadell K, Lindgren H, Tistad M (2017). Internet-based support for self-management strategies for people with COPD–protocol for a controlled pragmatic pilot trial of effectiveness and a process evaluation in primary healthcare. BMJ Open.

[63] Feldman HI, Appel LJ, Chertow GM, Cifelli D, Cizman B, Daugirdas J (2003). The Chronic Renal Insufficiency Cohort (CRIC) Study: Design and Methods. J Am Soc Nephrol.

